# Supplementation with vitamin D and its analogs for treatment of endothelial dysfunction and cardiovascular disease

**DOI:** 10.1590/1677-5449.190150

**Published:** 2020-07-06

**Authors:** Felipe Esdras Lucas Cardoso, Leandro da Cruz Melgaço dos Santos, Adirlene Pontes de Oliveira Tenório, Matheus Rodrigues Lopes, Romero Henrique de Almeida Barbosa

**Affiliations:** 1 Universidade Federal do Vale do São Francisco – UNIVASF, Campus Paulo Afonso, Paulo Afonso, BA, Brasil.

**Keywords:** vitamin D, cardiovascular diseases, endothelium, dietary supplements

## Abstract

Vitamin D (1,25-dihydroxycolecalciferol) is a prohormone that has attracted the interest of researchers since studies have shown that its effects are not restricted to bone metabolism. Thus, the present review summarizes the most recent findings and discusses the usefulness of prescribing vitamin D and its analogues for treatment and prevention of cardiovascular disorders and endothelial dysfunction. The paper constitutes a narrative review of the literature, selecting articles published from 2012 to 2019. Studies have shown that vitamin D3 and its analogues have beneficial effects on endothelial function, but these results are controversial, since research with larger samples and of longer duration found no reduction in morbidity and mortality or cardiovascular risk factors after use of these substances. Given the current state of the art, there is no clear scientific basis for supplementation with vitamin D or its analogues for treatment of endothelial dysfunction or cardiovascular disease.

## INTRODUCTION

Researchers have become interested in vitamin D (1,25-dihydroxycolecalciferol) in recent years, since studies demonstrated that its effects are not limited to bone metabolism. It is known that the receptors for this compound are found in several cell types, including endothelial cells. Since the pathogenesis of cardiovascular diseases involves changes to endothelium homeostasis, several hypotheses have been raised, leading to a variety of research efforts.

Considering that vitamin D deficiency is a risk factor for development of endothelial dysfunction,^1^ several studies have investigated the utility of supplementation with vitamin D and its analogues for treatment and prevention of conditions such as hypertension, myocardial infarction, and cerebrovascular disease, among others. This review summarizes the most recent findings on the subject and, based on the results of the research reviewed, discusses the utility of prescribing vitamin D and its analogues in clinical practice.

## METHODS

This paper is a narrative, bibliographic, review of the literature. Searches were run on the PubMed, SciELO, and LILACS databases. Narrative and systematic review articles, original articles, clinical trials, and case reports published from 2013 to 2019 in literature were selected using the following keywords: endothelial function, vitamin D, physiology, cardiovascular disease.

## DISCUSSION

### Physiological aspects

Vitamin D is a prohormone, i.e., it is biologically inactive, and action of solar ultraviolet radiation on 7-dehydrocholesterol is needed to activate it.^2^ Two hydroxylation reactions are needed to form the active compound. The first takes place in the liver, forming 25-hydroxyvitamin D (25-OHD3), also known as calcidiol. The second hydroxylation reaction takes place in the kidneys and forms the two principal metabolites, 1α,25-dihydroxyvitamin D [1α,25-(OH)2D3], known as calcitriol, and 24R,25-dihydroxyvitamin D3 [24R,25(OH)2D3], also known as 24-hydroxycalcidiol.^3^

**Figure 1:**
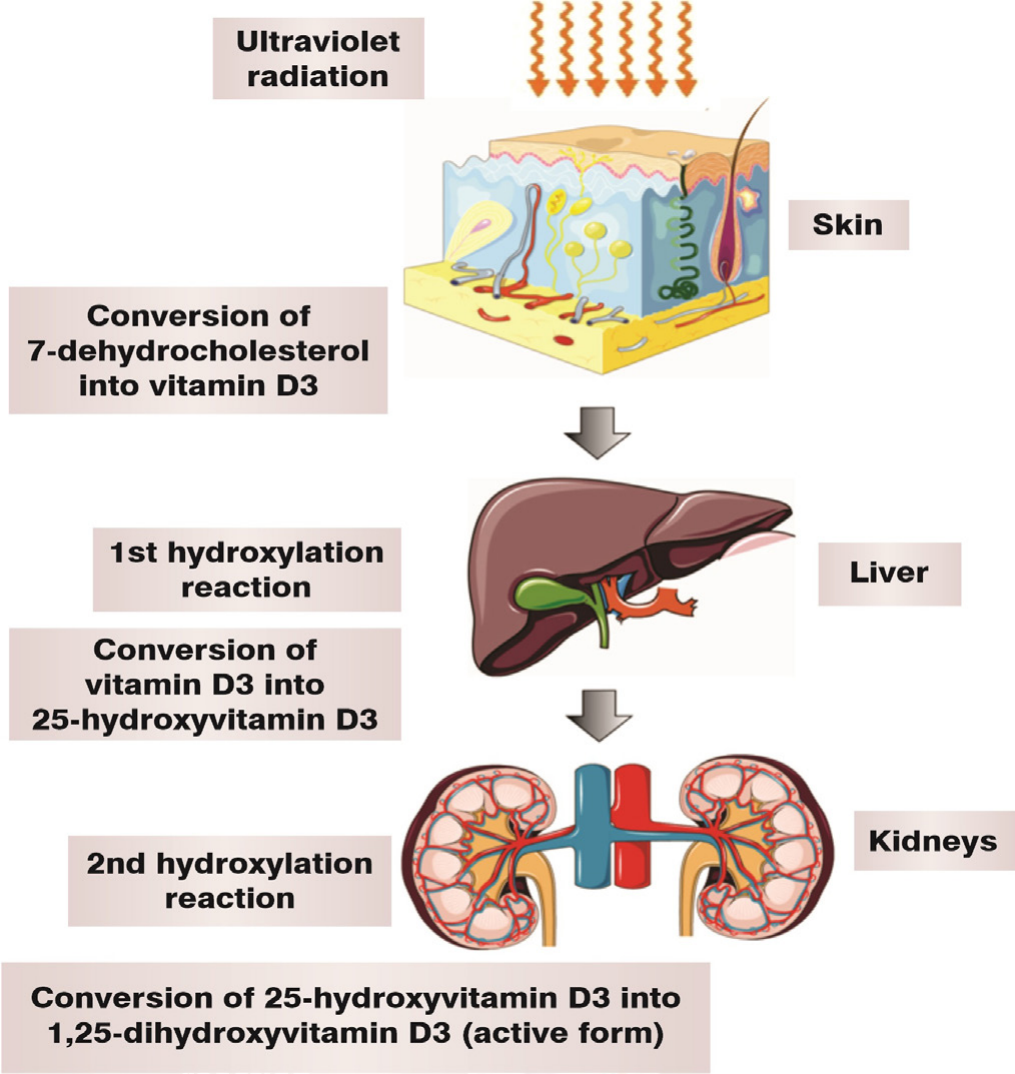
Vitamin D metabolism.

The kidney is the most important site involved in endocrine regulation of vitamin D, which occurs through rigorous control of the activity of the 1-hydroxylase enzyme. Production of calcitriol is modulated according to calcium concentrations and other endocrine requirements of the body. The primary factor that regulates production is the concentration of circulating calcitriol, which undergoes up-regulation by parathormone (PTH) and down-regulation by serum concentrations of calcium, phosphorus, and FGF23 (fibroblast growth factor), while calcitriol can be produced in many other tissues in the body^4^^,^^5^ (Figure 1).

### Actions of vitamin D

One of the most important actions of calcitriol is related to calcium homeostasis. In the intestine, it is responsible for stimulating calcium absorption through facilitated diffusion. In turn, renal resorption of calcium is also stimulated by 1,25(OH2)D3, more precisely in the distal tubules of the glomeruli, in a similar manner to intestinal absorption. Another phenomenon that is influenced by calcitriol is metabolism of bones, which constitute the largest store of calcium in the body and which use this ion to confer resistance on the skeleton. Therefore, absorption and resorption of calcium in the intestine and kidneys, respectively, are related to maintenance and integrity of bone structures.^5^

Recent research conducted with mice without the *Vdr* gene (which codes for the vitamin D receptor) and the *Cyp27B1* gene (which codes for alpha-1-hydroxylase) demonstrated that these animals had high levels of renin and, consequently, of angiotensin II, provoking hypertension and cardiac hypertrophy. It was also demonstrated that supplementation of healthy individuals with vitamin D3 provoked an increase in angiogenic myeloid cells, which play a role in vascular regeneration.^6^ Furthermore, cross-sectional studies with human beings indicated an inverse relationship between 25(OH)D3 levels and risk of hypertension.^5^ It therefore became clear that calcitriol is of fundamental importance for cardiovascular physiology, which sparked researchers’ interest in supplementation with vitamin D for treatment and prevention of cardiovascular diseases.

### Endothelial function

Endothelium is metabolically active tissue formed by a layer of endothelial cells with endocrine, autocrine, and paracrine functions.^7^ It is capable of modeling both the vascular lumen and the adjacent compartment of the smooth vascular musculature, by production of antiproliferative substances.^8^ The endothelium plays a protective role in blood vessels. This action is triggered by shear stress exerted by the blood flow on endothelial cells, resulting in low-level nitric oxide production, maintaining the blood vessel in a constant state of vasodilation.^7^ Nitric oxide is the principal substance responsible for endothelium-dependent vascular dilatation. Furthermore, it inhibits proliferation of smooth muscle cells, recruitment, adhesion, and differentiation of inflammatory cells, platelet aggregation, and production of thrombogenic thromboplastin,^9^ and also has an influence on reduction of expression of several inflammatory mediators.^10^

By activating vitamin D receptors (VDR) in endothelial cells, vitamin D provokes expression of vascular endothelial growth factor (VEGF). This important angiogenic factor acts on VEGF receptors, altering several cell activities, such as cell proliferation and survival, vascular permeability, and others. In turn, VEGF signaling is also involved in several cardiovascular diseases, mediating processes such as hypertrophic cardiomyopathies and formation of atherosclerotic plaques.^11^

**Figure 2:**
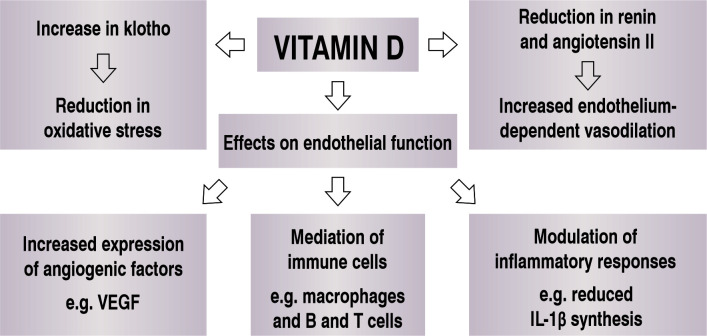
Action of vitamin D on endothelial function. IL = interleukin; VEGF = vascular endothelial growth factor.

It is known that the active form of vitamin D can be synthesized in endothelial cells by activity of specific α-hydroxylase. The product, 1,25(OH2)D3 acts on inflammatory mediators, modulating the activity of immune system cells such as macrophages, monocytes, and B and T lymphocytes.^12^ Furthermore, exposure of the active form of vitamin D to endothelial cells reduces expression of proinflammatory substances, such as IL-1β, which is inversely related to normal endothelial function.^13^ There is thus a clear relationship between vitamin D physiology and normal endothelium function and this substance is also involved in the pathogenesis of several cardiovascular diseases (Figure 2).

### Repercussions of supplementation with calcitriol and its analogues for endothelial function

It has been demonstrated in vitro that vitamin D is involved in protection against oxidative stress in a study using endothelial cells from human umbilical veins, in which samples of these cells were exposed to vitamin D for 24 hours before exposure to oxidative stress caused by H_2_O_2_ and compared to samples that were not exposed to vitamin D. The group of cells treated with vitamin D was protected from the oxidative stress mediated by the superoxide anion. Furthermore, it was also observed that apoptosis mediated by cascade activation was inhibited. Vitamin-D-mediated MEK/ERK/SirT-1 axis activation was also observed, reducing endothelial injury and dysfunction caused by oxidative stress.^14^

The action of calcitriol on renovascular function was assessed in vitro after exposure of renal arteries to calcitriol, observing increased renal arterial dilatation and reduced expression of enzymes related to oxidative stress, such as NOX-2, NOX-4, and others. There was also a reduction in endothelium-dependent contractions.^15^

Paricalcitol is a non-hypercalcemic vitamin D analogue. Its effects were analyzed in a model of acute kidney injury induced in mice by ischemia-reperfusion. It is known that kidney injury involves complex relations between damage to tubule cells, inflammation, and endothelial dysfunction. In this study, one group of mice was pre-treated with paricalcitol, 1 day before ischemia. Another group was given the same volume of a vehicle. After testing, it was concluded that the animals treated with paricalcitol exhibited attenuation of renal injury and inflammation, manifest as lower levels of cytokines and reduced infiltration of leukocytes in the kidneys.^16^

Takenaka et al.^17^ evaluated vitamin D’s potential for suppression of oxidative stress using four groups of hypertensive rats: controls (C); rats treated with irbesartan (I); rats treated with calcitriol (V); and rats treated with irbesartan and calcitriol (I + V). The group treated with irbesartan and calcitriol (I + V) exhibited attenuation of albuminuria and reduced concentrations of renal angiotensin II. The advantages observed after treatment with calcitriol only included lower plasma angiotensin II levels and increased klotho expression. This substance has antioxidant effects, because it induces production of superoxide dismutase, which is an important enzyme in protection against the harmful effects of oxygen species.

The effects of vitamin D on the renin-angiotensin-aldosterone system were assessed in a study comparing essential hypertensive patients with hypovitaminosis D, essential hypertensive patients with normal vitamin D levels, and normotensive individuals. When the individuals with hypertension and hypovitaminosis D were given supplementation with cholecalciferol for 8 weeks, they exhibited reductions in plasma renin levels and increases in blood flow-mediated vasodilation.^18^

In addition to vitamin D deficiency, obesity and overweight are important risk factors related to development of endothelial dysfunction. Based on this fact, Borgi et al.^19^ conducted a randomized, double-blind, placebo-controlled study with obese and overweight individuals free from hypertension and diabetes. The participants were given ergocalciferol or placebo. At the end of the study, no significant change in endothelium-dependent dilatation was observed in the group given ergocalciferol in relation to the group given placebo.

A randomized, controlled trial assessed the impact of vitamin D3 supplementation on 200 hypertensive participants with 25-hydroxyvitamin D levels below 30 ng/ml. A group of 100 people who were given vitamin D3 during the trial was compared to a group of 100 individuals given placebo only. The primary parameter assessed was systolic pressure at 24 hours; secondary parameters included diastolic pressure at 24 hours, and levels of renin, aldosterone, and the N-terminal portion of the prohormone type B natriuretic peptide (NT-proBNP), QT interval corrected by heart rate, 24-hour urinary albumin excretion, and others. One hundred and eight patients completed the trial, but no significant beneficial effects of vitamin D3 on arterial blood pressure or other cardiovascular risk factors were observed.^20^

This finding was consistent with the results of the DAYLIGHT trial, which investigated the effects of vitamin D supplementation on blood pressure levels in hypertense and pre-hypertensive patients. A total of 383 patients completed the 6-month study, but the group given high doses of supplementation did not exhibit significant reductions in mean 24-hour systolic pressure in comparison to the group administered lower doses.^21^

Recently, a randomized, placebo-controlled study compared the effects of administration of vitamin D (2000 UI/day) for prevention of cardiovascular diseases and cancer against administration of placebo only. The study lasted 5 years and involved 25,871 people and demonstrated that the incidence of cardiovascular events (myocardial infarction, stroke, and death from cardiovascular causes) was not significantly lower in the group given the vitamin than in the group given placebo. Along the same lines, there was no reduction in the incidence of deaths from cancer in the group given vitamin D.^22^

More conclusive data on the efficacy of supplementation with vitamin D for prevention of cardiovascular diseases were obtained in a meta-analysis conducted by Barbarawi et al.^23^ This review analyzed 21 randomized clinical trials with more than 83,000 participants to determine the possible efficacy of supplementation with vitamin D for reduction of cardiovascular events. No significant reductions were observed in cardiovascular or cerebrovascular events or in mortality from these conditions.

## CONCLUSIONS

The need to maintain vitamin D at physiological levels in the body has been well-established in the literature, since hypovitaminosis is related to the risk of developing endothelial dysfunction.^24^ Although several studies have identified beneficial effects of vitamin D and its analogues on endothelial function and aspects directly linked to it, these results are controversial. Recent studies with large samples and long duration did not detect significant improvements in endothelial function or cardiovascular risk factors after use of these substances (Table 1).^6^^,^^14^^-^23

**Table 1:** Data on the studies included in the literature review.

**Study**	**Type of study**	**Methods**	**Main findings**
Dong et al.^15^ - Hong Kong	Study with endothelial cells from human or rat aorta	Exposure of renal arteries to calcitriol or angiotensin II	In vivo and in vitro activation of the vitamin D receptor with calcitriol improves endothelial function
Polidoro et al.^14^ - Italy	In vitro study	Administration of vitamin D to human umbilical vein endothelial cells	Protection against oxidative stress, mediated by superoxide
Lee et al.^16^ - Republic of Korea	In vivo with mice and in vitro with HK-2 cells	Evaluation of renal inflammation and injury and the direct effect of paricalcitol on tubule cells	Renoprotective effect in acute ischemic kidney injury
Takenaka et al.^17^ - Japan	Experimental study with hypertensive rats	Hypertensive, uninephrectomized rats, treated with vitamin D	Improved expression of klotho and suppression of oxidative stress and albuminuria, without substantial changes to renal angiotensin II levels
Wong et al.^6^ - Germany	In vivo and in vitro study	Supplementation with vitamin D3 in healthy donors and mice	Improved vascular regeneration after injury in healthy and diabetic individuals
Pilz et al.^20^ - Germany	Randomized clinical trial, double-blind, placebo-controlled	Supplementation with vitamin D3 for 8 weeks with 200 hypertensive participants with low 25-hydroxyvitamin D levels	No significant beneficial effect of vitamin D3 on arterial blood pressure or other cardiovascular risk factors was observed, but it was associated with a significant increase in triglycerides
Carrara et al.^18^ - Italy	Clinical case-control study	Thirty-three patients with essential hypertension and hypovitaminosis D were treated with cholecalciferol for 8 weeks	Restoration of normal vitamin D levels is capable of inhibiting the renin-angiotensin system and improving flow-mediated dilation
Borgi et al.^19^ - United States	Randomized, double-blind, placebo-controlled, clinical trial	Forty-six nonhypertensive, nondiabetic overweight, or obese individuals with vitamin D deficiency were given ergocalciferol or matching placebo for 8 weeks	No improvement in endothelial function after vitamin D replacement
Arora et al.^21^ - United States	Multicenter study, randomized, double-blind	Vitamin D supplementation at high or low doses in 534 hypertensive or pre-hypertensive individuals with vitamin D deficiency,	No significant reductions in mean 24-hour systolic pressure
Manson et al.^22^ - United States	Randomized, placebo-controlled, clinical trial,	Administration of vitamin D3 and marine omega 3 fatty acids to a total of 25,871 participants, for primary prevention of cancer and cardiovascular diseases	Supplementation with vitamin D did not result in lower incidence of invasive cancer or cardiovascular events when compared with placebo
Barbarawi et al.^23^ - United States	Meta-analysis of 21 randomized clinical trials	Efficacy of supplementation with vitamin D for reduction of cardiovascular events and all-causes mortality, including 83,291 patients, 41,669 of whom were given vitamin D and 41,622 of whom were given placebos	No significant reductions were observed in cardiovascular events, cerebrovascular events, or mortality

We therefore conclude that there is no clear scientific basis for supplementation with vitamin D or its analogues for treatment of endothelial dysfunction or cardiovascular diseases. It should be emphasized, however, that there is still a need for more extensive research to elucidate the subject further, thereby providing health professionals with greater certainty on the need for vitamin D supplementation.
