# Supplementary material for: Supplementation with vitamin D and its analogs for treatment of endothelial dysfunction and cardiovascular disease
Source: J Vasc Bras. 2020 Jul 6;19:e20190150. doi: 10.1590/1677-5449.190150 (PMC8202173; doi:10.1590/1677-5449.190150)
Supplement: Translation

# Translation: Suplementação de vitamina D e seus análogos para tratamento de disfunção endotelial e doenças cardiovasculares

**Authors:** Felipe Esdras Lucas Cardoso, Leandro da Cruz Melgaço dos Santos, Adirlene Pontes de Oliveira Tenório, Matheus Rodrigues Lopes, Romero Henrique de Almeida Barbosa

**Affiliations:** 1 Universidade Federal do Vale do São Francisco – UNIVASF, Campus Paulo Afonso, Paulo Afonso, BA, Brasil.

**Keywords:** vitamina D, doenças cardiovasculares, endotélio, suplementos nutricionais

## Abstract

A vitamina D (1,25-dihidroxicolecalciferol) é um pró-hormônio que tem despertado a atenção de pesquisadores após estudos demonstrarem que seus efeitos não estão restritos ao metabolismo ósseo. Assim, a presente revisão sintetiza os achados mais recentes e discute a utilidade da prescrição de vitamina D e seus análogos no tratamento e prevenção de afecções cardiovasculares e disfunção endotelial. Este trabalho consiste em uma revisão narrativa da literatura feita a partir da seleção de artigos publicados no período de 2012 a 2019. Estudos demonstraram efeitos benéficos da vitamina D3 e seus análogos sobre a função endotelial; no entanto, tais resultados mostram-se controversos, visto que pesquisas com maior amostragem e duração não encontraram redução na morbimortalidade ou nos fatores de risco cardiovascular após o uso de tais substâncias. Frente ao estado atual da arte, não existe embasamento científico claro para suplementação de vitamina D ou seus análogos para tratamento de disfunção endotelial ou doenças cardiovasculares.

## INTRODUÇÃO

A vitamina D (1,25-dihidroxicolecalciferol) tem despertado a atenção de pesquisadores nos últimos anos, após estudos demonstrarem que seus efeitos não estão restritos ao metabolismo ósseo. Sabe-se que os receptores desse composto estão presentes em vários tipos celulares, incluindo células endoteliais. Uma vez que a patogênese de doenças cardiovasculares envolve alterações na homeostase do endotélio, algumas hipóteses foram propostas e levaram ao desenvolvimento de variadas pesquisas.

Considerando que a deficiência de vitamina D é um fator de risco para o desenvolvimento de disfunção endotelial^1^, muitos estudos abordaram a utilidade da suplementação da vitamina D e seus análogos no tratamento e prevenção de afecções como hipertensão, infarto do miocárdio, doença cerebrovascular, entre outras. Assim, a presente revisão sintetiza os achados mais recentes a respeito do tema e, de acordo com os resultados das pesquisas, discute a utilidade da prescrição de vitamina D e seus análogos na prática clínica.

## MÉTODOS

O presente trabalho é uma revisão bibliográfica narrativa da literatura. As buscas foram realizadas nas bases de dados PubMed, SciELO e LILACS. Na pesquisa, foram selecionados artigos de revisões narrativas e sistemáticas, artigos originais, ensaios clínicos e relatos de casos do período de 2013 a 2019 na literatura, utilizando as seguintes palavras-chave: função endotelial, vitamina D, fisiologia e doença cardiovascular.

## DISCUSSÃO

### Aspectos fisiológicos

A vitamina D é um pró-hormônio, ou seja, é biologicamente inativa, sendo necessário que ocorra ação da radiação ultravioleta solar sobre o 7-deidrocolesterol para sua ativação^2^. São necessárias duas hidroxilações para formação do composto ativo: a primeira ocorre no fígado, formando a 25-hidroxivitamina D (25-OHD3), denominada calcidiol. A segunda hidroxilação ocorre nos rins e forma seus dois metabólitos principais, a 1α,25-dihidroxivitamina D [1α,25-(OH)2D3], conhecida como calcitriol, e o 24R,25-dihidroxivitamina D3 [24R,25(OH)2D3], também conhecido como 24-hidroxicalcidiol^3^.

O rim é o local mais importante na regulação endócrina da vitamina D, a qual ocorre através do controle rigoroso da atividade da enzima 1-hidroxilase. A produção do calcitriol é modulada conforme as concentrações de cálcio e outras necessidades endócrinas do organismo. Os principais fatores reguladores da produção são a concentração do calcitriol circulante, sofrendo *up-regulation* pelo paratormônio (PTH) e *down-regulation* pelas concentrações séricas de cálcio, fósforo e FGF23 (fator de crescimento de fibroblastos), podendo o calcitriol ser produzido em diversos outros tecidos do organismo^4^^,^^5^ (Figura 1).

**Figura 1:** Esquema do metabolismo da vitamina D.

### Ações da vitamina D

Uma das principais ações do calcitriol está relacionada à homeostase do cálcio. No intestino, ele é responsável por estimular a absorção de cálcio através da difusão facilitada. Por sua vez, a reabsorção renal de cálcio também é estimulada pelo 1,25(OH2)D3, mais precisamente nos túbulos distais dos glomérulos, de forma semelhante à absorção intestinal. Outro aspecto influenciado pelo calcitriol é o metabolismo dos ossos, que formam o maior reservatório de cálcio no organismo e utilizam esse íon para conferir resistência ao esqueleto. Dessa forma, a absorção e reabsorção de cálcio no intestino e rins, respectivamente, estão relacionadas à manutenção da integridade das estruturas ósseas^5^.

Pesquisas recentes com camundongos sem os genes *Vdr* (que codifica o receptor de vitamina D) e *Cyp27B1* (que codifica a alfa-1-hidroxilase) demonstraram que esses animais possuíam altos níveis de renina e, consequentemente, de angiotensina II, provocando hipertensão e hipertrofia cardíaca. Foi demonstrado que a suplementação de vitamina D3 em indivíduos saudáveis promoveu o aumento de células mieloides angiogênicas, que desempenham papel na regeneração vascular^6^. Além disso, estudos transversais com seres humanos indicaram relação inversa entre níveis de 25(OH)D3 e risco de hipertensão^5^. Dessa maneira, percebe-se que o calcitriol possui fundamental importância na fisiologia cardiovascular, o que despertou o interesse de pesquisadores a respeito da suplementação de vitamina D para tratamento e prevenção de doenças cardiovasculares.

### Função endotelial

O endotélio é um tecido metabolicamente ativo formado por uma camada de células endoteliais com funções endócrinas, autócrinas e parácrinas^7^. Ele tem a capacidade de modular tanto o lúmen vascular como o compartimento adjacente da musculatura lisa vascular, pela produção de substâncias antiproliferativas^8^. O endotélio desempenha papel protetor do vaso sanguíneo. Essa ação acontece através de tensão de cisalhamento, ou *shear stress,* exercido pelo fluxo sanguíneo sobre as células endoteliais, que resulta na formação basal de óxido nítrico, mantendo o vaso sanguíneo em um estado constante de vasodilatação^7^. O óxido nítrico é a principal substância responsável pela dilatação vascular dependente do endotélio. Além disso, inibe a proliferação das células musculares lisas, o recrutamento, a adesão e a diferenciação de células inflamatórias, a agregação plaquetária e a produção do fator tecidual trombogênico^9^, influenciando também na redução da expressão de vários mediadores inflamatórios^10^.

A vitamina D, ao ativar o receptor de vitamina D (VDR) nas células endoteliais, promove a expressão de fator de crescimento endotelial vascular (VEGF). Esse importante fator angiogênico age sobre os receptores de VEGF, alterando várias atividades celulares, como proliferação e sobrevivência celulares, permeabilidade vascular, entre outras. A sinalização do VEGF, por sua vez, também está envolvida em diversas doenças cardiovasculares, mediando processos como cardiomiopatias hipertróficas e formação de placas ateroscleróticas^11^.

Sabe-se que a forma ativa da vitamina D pode ser sintetizada em células endoteliais, por meio da ação de α-hidroxilase específica. O produto, 1,25(OH2)D3, possui atividade sobre mediadores inflamatórios, modulando a ação de células do sistema imune como macrófagos, monócitos e linfócitos B e T^12^. Além disso, a exposição da forma ativa da vitamina D a células endoteliais diminui a expressão de substâncias pró-inflamatórias, como IL-1β, que está inversamente relacionada à função endotelial normal^13^. Assim, é possível perceber a relação entre a fisiologia da vitamina D e sua relação com a função normal do endotélio bem como o envolvimento dessa substância na patogênese de diversas doenças cardiovasculares (Figura 2).

**Figura 2:** Ação da vitamina D na função endotelial. IL = interleucina; VEGF = fator de crescimento endotelial vascular.

### Repercussões da suplementação de calcitriol e seus análogos na função endotelial

Foi demonstrado, *in vitro,* o envolvimento da vitamina D na proteção ao estresse oxidativo em um estudo com células endoteliais da veia umbilical humana, no qual parte das células foi exposta a vitamina D por 24 horas e, posteriormente, a estresse oxidativo causado por H_2_O_2_, enquanto outra parte não foi exposta a vitamina D. O grupo de células tratado com a vitamina D foi protegido desse estresse oxidativo mediado por ânion superóxido. Além disso, foi observado que a apoptose mediada pela ativação da cascata foi inibida. Também foi notada a ativação do eixo MEK/ERK/SirT-1 mediada pela vitamina D, que reduziu disfunção e lesão endoteliais causadas pelo estresse oxidativo^14^.

A atividade do calcitriol sobre a função renovascular foi avaliada *in vitro* após a exposição de artérias renais a calcitriol, havendo aumento da dilatação arterial renal além de menor expressão de enzimas relacionadas ao estresse oxidativo, como NOX-2, NOX-4, entre outras. Também houve diminuição das contrações endotélio-dependentes^15^.

O paricalcitol, análogo de vitamina D não hipercalcêmico, teve seus efeitos avaliados num modelo de lesão renal aguda induzida por isquemia/reperfusão em camundongos. Sabe-se que a lesão renal abrange relações complexas entre dano das células tubulares, inflamação e disfunção endotelial. Nesse contexto, um grupo de camundongos foi pré-tratado com paricalcitol 1 dia antes da isquemia. Outro grupo recebeu o mesmo volume de veículo. Após os testes, concluiu-se que os animais tratados com paricalcitol apresentaram atenuação da injúria e da inflamação renais por meio da diminuição dos níveis de citocinas e da infiltração de leucócitos nos rins^16^.

Takenaka et al.^17^ avaliaram o potencial da vitamina D na supressão do estresse oxidativo usando quatro grupos de ratos hipertensos: controles (C); tratados com irbesartana (I); tratados com calcitriol (V); e tratados com irbesartana e calcitriol (I + V).O grupo tratado com irbesartana e calcitriol (I + V) apresentou atenuação da albuminúria e redução da concentração de angiotensina II renal. As vantagens do tratamento apenas com calcitriol incluíram menores níveis de angiotensina II plasmática e aumento de klotho. Essa substância apresenta efeitos antioxidantes, pois induz a produção de superóxido dismutase, uma enzima importante na proteção contra efeitos lesivos das espécies de oxigênio.

Os efeitos da vitamina D sobre o sistema renina-angiotensina-aldosterona foram também avaliados em um estudo comparativo entre pacientes hipertensos essenciais com hipovitaminose D, pacientes hipertensos essenciais com níveis normais de vitamina D e indivíduos normotensos. Os indivíduos com hipertensão e hipovitaminose D, ao serem submetidos à suplementação com colecalciferol por 8 semanas, apresentaram redução dos níveis de renina plasmática e aumento na vasodilatação mediada por fluxo sanguíneo^18^.

Além da deficiência de vitamina D, a obesidade e o sobrepeso figuram como importantes fatores de risco relacionados ao desenvolvimento de disfunção endotelial. Baseados nesse fato, Borgi et al.^19^ conduziram um estudo randomizado, duplo-cego e placebo-controlado com indivíduos obesos e com sobrepeso, não hipertensos e não diabéticos. Os participantes receberiam ergocalciferol ou placebo. Ao final do estudo, não foi percebida mudança significativa na dilatação endotélio-dependente no grupo que recebeu ergocalciferol em relação ao grupo que recebeu placebo.

Um ensaio randomizado controlado avaliou o impacto da suplementação de vitamina D3 sobre 200 participantes hipertensos e com níveis de 25-hidroxivitamina D abaixo de 30 ng/ml. O grupo das 100 pessoas que receberam vitamina D3 durante o ensaio foi comparado ao grupo dos 100 indivíduos restantes que receberam apenas placebo. O parâmetro primário de avaliação foi a pressão sistólica em 24 horas; os parâmetros secundários incluíram pressão diastólica em 24 horas, níveis de renina, aldosterona e de porção N-terminal do pró-hormônio do peptídeo natriurético do tipo B (NT-proBNP), intervalo QT corrigido pela frequência cardíaca, excreção urinária de albumina em 24 horas, entre outros. Cento e oitenta e oito pacientes completaram o ensaio, no qual não foi observado efeito benéfico significante da vitamina D3 sobre a pressão arterial e sobre outros fatores de risco cardiovasculares^20^.

Esse achado foi consistente com os resultados do ensaio *DAYLIGHT*, que observou os efeitos da suplementação de vitamina D sobre níveis pressóricos de pacientes hipertensos e pré-hipertensos. Dos 383 pacientes que completaram o estudo de 6 meses, o grupo que recebeu altas doses de suplementação não apresentou reduções significativas na pressão sistólica média de 24 horas, em relação ao grupo ao qual foram administradas doses mais baixas^21^.

Recentemente, um estudo randomizado e placebo-controlado comparou os efeitos da administração de vitamina D (2000 UI/dia) sobre a prevenção de doenças cardiovasculares e câncer com a mera administração de placebo. A pesquisa, que durou 5 anos e envolveu 25.871 pessoas, demonstrou que não houve incidência significativamente menor de eventos cardiovasculares (infarto do miocárdio, acidente vascular encefálico e morte por causas cardiovasculares) no grupo que recebeu a substância quando comparado ao grupo que recebeu placebo. Do mesmo modo, não houve redução na incidência de mortes por câncer no grupo que recebeu vitamina D^22^.

Dados mais conclusivos sobre a eficiência da suplementação de vitamina D na prevenção de doenças cardiovasculares foram obtidos através de metanálise conduzida por Barbarawi et al.^23^. Nesse trabalho, 21 ensaios clínicos randomizados, que incluíram mais de 83.000 participantes, foram analisados quanto à possível eficácia da suplementação de vitamina D na redução de eventos cardiovasculares. Não foi observada diminuição significativa em eventos cardiovasculares ou cerebrovasculares nem na mortalidade por tais morbidades.

## CONCLUSÃO

É bem descrita na literatura a necessidade de manter níveis fisiológicos normais de vitamina D no organismo, uma vez que a hipovitaminose está relacionada ao risco de desenvolver disfunção endotelial^24^. Apesar de diversos estudos apontarem ações benéficas da vitamina D e de seus análogos sobre a função endotelial e aspectos diretamente ligados a ela, esses resultados são controversos. Pesquisas recentes, de grande amostragem e duração, não encontraram melhoras significativas na função endotelial ou em fatores de risco cardiovasculares após uso de tais substâncias (Tabela 1)^6^^,^^14^^-^23.

**Tabela 1:** Dados dos estudos incluídos na revisão de literatura.

**Estudo**	**Tipo de estudo**	**Métodos**	**Resultados principais**
Dong et al.^15^ - Hong Kong	Estudo com células endoteliais da aorta humana ou de rato	Exposição de artérias renais ao calcitriol ou à angiotensina II	A ativação *in vivo* e *in vitro* do receptor de vitamina D com calcitriol melhora a função endotelial
Polidoro et al.^14^ - Itália	Estudo *in vitro*	Administração de vitamina D em células endoteliais da veia umbilical humana	Proteção contra estresse oxidativo, mediada por superóxido
Lee et al.^16^ - República da Coréia	Estudo *in vivo* com camundongos e *in vitro* com células HK-2	Avaliação da lesão e inflamação renal e do efeito direto do paricalcitol nas células tubulares	Efeito renoprotetor na lesão renal aguda isquêmica
Takenaka et al.^17^ - Japão	Estudo experimental com ratos hipertensos	Ratos hipertensos, uninefrectomizados e tratados com vitamina D	Melhora na expressão do klotho e supressão do estresse oxidativo e da albuminúria sem alterações substanciais nos níveis renais de angiotensina II
Wong et al.^6^ - Alemanha	Estudo *in vivo* e *in vitro*	Suplementação com vitamina D3 em doadores saudáveis e em camundongos	Melhora da regeneração vascular após lesão em indivíduos saudáveis e diabéticos
Pilz et al.^20^ - Alemanha	Ensaio clínico randomizado, duplo-cego, controlado com placebo	Suplementação de vitamina D3 por 8 semanas com 200 participantes hipertensos e com níveis de 25-hidroxivitamina D baixos	Não foi observado efeito benéfico significante da vitamina D3 sobre a pressão arterial e outros fatores de risco cardiovasculares, mas foi associada a um aumento significativo de triglicerídeos
Carrara et al.^18^ – Itália	Estudo clínico caso-controle	Trinta e três pacientes com hipertensão essencial e hipovitaminose D foram submetidos à terapia com colecalciferol por 8 semanas	Restauração dos níveis normais de vitamina D é capaz de inibir o sistema renina-angiotensina e melhorar a dilatação mediada por fluxo
Borgi et al.^19^ - Estados Unidos	Ensaio clínico randomizado, duplo-cego, controlado com placebo	Quarenta e seis indivíduos não hipertensos, com sobrepeso não diabéticos ou obesos com deficiência de vitamina D receberam ergocalciferol ou placebo correspondente durante 8 semanas	Não houve melhora na função endotelial após a reposição de vitamina D
Arora et al.^21^ - Estados Unidos	Estudo multicêntrico, randomizado, duplo-cego	Suplementação de vitamina D em altas ou baixas doses em 534 indivíduos com deficiência de vitamina D, hipertensos ou pré-hipertensos	Sem reduções significativas na pressão sistólica média de 24 horas
Manson et al.^22^ - Estados Unidos	Ensaio clínico randomizado, controlado com placebo	Administração de vitamina D3 e ácidos graxos ômega-3 marinhos em um total de 25.871 participantes, para prevenção primária de câncer e doenças cardiovasculares	A suplementação com vitamina D não resultou em menor incidência de câncer invasivo ou eventos cardiovasculares quando comparada ao placebo
Barbarawi et al.^23^ - Estados Unidos	Metanálise de 21 ensaios clínicos randomizados	Eficácia da suplementação de vitamina D na redução de eventos cardiovasculares e mortalidade por todas as causas, incluindo 83.291 pacientes, dos quais 41.669 receberam vitamina D e 41.622 receberam placebos	Não foi observada diminuição significativa em eventos cardiovasculares ou cerebrovasculares nem na mortalidade

Assim, concluímos não haver embasamento científico claro para suplementação de vitamina D ou de seus análogos no tratamento de disfunção endotelial ou doenças cardiovasculares. Vale ressaltar, no entanto, que ainda existe a necessidade de maiores pesquisas sobre o tema para melhor elucidar a questão e, assim, permitir maior certeza aos profissionais de saúde quanto à necessidade de suplementação de vitamina D.
